# Health interventions do not cause Malthusian conflict dynamics: Evidence from the expansion of HIV treatment in Africa

**DOI:** 10.1371/journal.pgph.0005448

**Published:** 2026-08-19

**Authors:** Andrea Berlanda, Matteo Cervellati, Elena Esposito, Dominic Rohner, Uwe Sunde

**Affiliations:** 1 Department of Economics, University of Bergamo, Bergamo, Italy; 2 Department of Economics, University of Bologna, Bologna, Italy; 3 CEPR, London, United Kingdom; 4 ESOMAS Department, University of Torino, Torino, Italy; 5 Collegio Carlo Alberto, Torino, Italy; 6 Department of Economics, University of Lausanne, Lausanne, Switzerland; 7 Department of Economics, University of Munich (LMU), Munich, Germany; Islamic Azad University South Tehran Branch, IRAN, ISLAMIC REPUBLIC OF

## Abstract

Recent research concludes that health improvements in the 20th century caused a surge in social conflict by accelerating population growth. Rationalized by Malthusian population dynamics, this indicates a potential trade-off for world-wide health initiatives, especially in conflict-prone regions. Here, we re-investigate this question with fine-grained contemporary data. After conceptualizing the chain of mechanisms that links health-driven population growth to conflicts, we address the question whether health improvements cause Malthusian conflict dynamics in 50 countries in Africa over the period 1990–2017. The analysis combines the massive worldwide roll-out of antiretroviral therapy (ART) for HIV-positive individuals during the early 2000s with local variation in treatment potential before the onset of this expansion. We find that the deployment of ART has indeed led to a demographic expansion. In sharp contrast to the literature on Malthusian dynamics, we find that this health-induced population growth has not resulted in a surge of conflict measured in terms of (small or medium scale) social violence or large scale armed conflict. For armed conflict, we find no effects, whereas for social violence we detect a reduction. The findings substantially qualify existing evidence by documenting that public health provision and medical innovation, while entailing accelerated population growth, do not necessarily induce Malthusian conflict dynamics, but rather may entail a “double dividend” in terms of health improvements and a reduction in conflicts.

## Author summary

In recent years, warnings of Malthusian conflict dynamics have become increasingly popular. The argument brought forward is that health improvements in poor countries may lead to population growth that could fuel social violence and armed conflicts through higher resource pressure. The current article draws on very fine-grained data for the expansion of antiretroviral therapy (ART) for HIV-positive individuals during the early 2000s to study the impact of better health treatment on population growth and, in turn, the effect of population growth on conflict in 50 African countries over the period 1990–2017. We find that better health care positively affects population growth, as expected. Yet, the link between population growth and conflict is conceptually more subtle. On the one hand, accelerated population growth may exacerbate resource scarcity and conflict, yet on the other hand, better health may reduce grievances by inducing economic development through scale effects, higher labor productivity and market expansion, which in turn could attenuate conflict risks. Empirically, the article finds that the expansion of ART treatment has —if anything—led to an overall reduction in social violence in Africa. These findings qualify existing arguments on Malthusian conflict dynamics and imply that rather than a trade-off, global health care investments may improve health and reduce conflict, thereby entailing a “double dividend”.

## Introduction

One of the most enduring scholarly debates concerns Malthus’ (1798) conjecture that population expansions unavoidably trigger conflicts and wars. Conceptually, Malthusian mechanisms are at work if the population expansion exceeds economic capacity and thereby leads to an increased scarcity of resources such as land or water [1]. Malthusian dynamics are considered an inadequate description of high-income countries, but population growth is still seen as a main obstacle for development in many low-income countries [2,3]. In view of this, some scholars have argued that diseases like AIDS, despite the tragedy of human suffering and the loss of lives, might lead to larger per capita consumption possibilities by curbing population growth in Africa [4]. While controversially debated, variants of these ideas have made a significant comeback in recent publications that provide evidence for a causal link from population growth to armed conflict. Population expansions related to exogenous factors like favorable meteorological conditions or commodity prices have been associated with more social conflict [5–7]. These findings have been associated with greater population density leading to greater scarcity of food and resources, especially in the context of economic stagnation. Similar arguments have been made in surveys of the conflict literature [8–10].

Recent work pushes this perspective by tracing the root cause of the rise in conflicts to health improvements. Better health in the context of medical innovation and its worldwide dissemination during the epidemiological transition of the mid-20th century was associated with population growth and more conflict [11]. Whether Malthusian conflict dynamics triggered by health interventions are at work today remains an open empirical question that is crucial to address: the policy implications derived from the existing (historical) evidence are hard to overstate as, if confirmed, they imply the existence of a serious trade-off for health interventions, particularly for today’s poorest, conflict-prone countries.

The present research reconsiders the question whether population dynamics due to health interventions lead to increased conflict. Compared to previous research, we apply a similar empirical methodology but we exploit fine-grained data on a recent major health intervention — the roll-out of antiretroviral therapy for HIV-positive individuals across the African continent. We find that health-induced population increases had no discernable impacts on (large-scale) armed conflict and significantly decreased (small and medium-scale) social violence.

## Background

**Fig 1:**
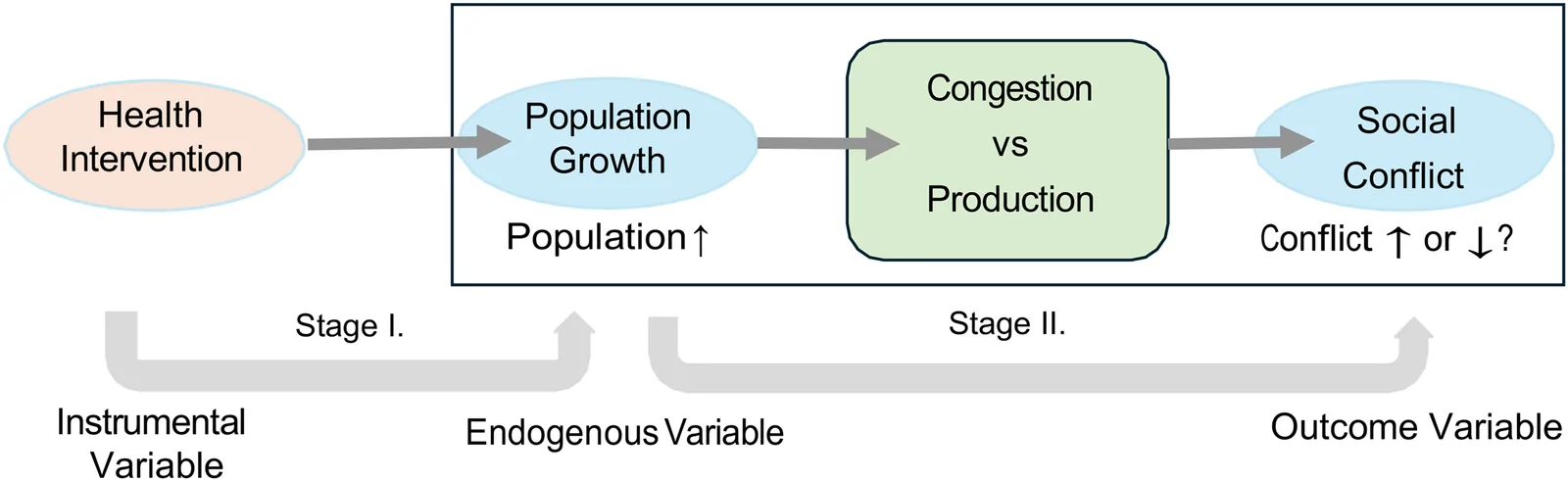
Conceptual framework.

Social conflict and population dynamics are closely intertwined, with causality running both ways. The focus of this paper is on isolating the Malthusian mechanism of population dynamics causing conflict. In theory, the effect of a health-driven acceleration of population growth on conflict is a priori ambiguous. Fig 1 illustrates the underlying conceptual framework. Stage I. of the argument states that health improvements lead to a mortality reduction and thereby, with fertility rates unchanged, to an acceleration of population growth. This effect is uncontroversial in light of the existing evidence and works through potentially various channels. For instance, health improvements increase population directly through lower mortality and indirectly through improved living conditions due to lower health costs and higher labor productivity. Stage II. of the argument links population growth to conflict outcomes. This link is less clear. On the one hand, accelerated population growth can exacerbate resource scarcity and conflict [12]; on the other hand, better health reduces existential pressure and grievances, thereby reducing the incentives to protest and raising the opportunity cost for engaging in conflict. Moreover, economies of scale and increases in productivity associated with better health may foster specialization, the development of markets, and technology adoption [13,14], thereby relaxing resource scarcity and boosting revenues from productive activities. As a result, the effect of health-driven population growth on conflicts is a priori ambiguous.

Empirical studies on the question of health-driven Malthusian conflict typically implement this conceptual framework using an instrumental variables approach. This approach makes use of health interventions or mortality changes that are plausibly exogenous to the prevalence of conflict and that serve as an instrumental variable.

Using this variation to predict the respective response of population dynamics allows isolating the causal effect of a Malthusian population expansion on conflict outcomes. This causal identification approach has been adopted in recent work that studied the effect of population dynamics on conflict using health improvements in the context of the global epidemiological transition during the middle of the 20th century [11].

Specifically, their study makes use of cross-sectional variation in mortality from various diseases prior to the invention of drugs and vaccines to treat, cure, or prevent these diseases, and then exploits the subsequent reduction in the mortality caused by these diseases during the epidemiological transition between 1940 and 1980 to predict population dynamics. The validity of these findings based on historical data at a global scale for conflicts today remains unclear, specifically in Africa, the continent exhibiting the fastest population growth and receiving the largest portion of foreign health interventions. This study therefore revisits the question of health-driven Malthusian conflict using a similar identification approach but more recent and fine-grained data for the early 2000s.

## Data and methods

### Ethics statement

The analysis is based on publicly available data. It is not based on personal information of human subjects and does not include interventions. Therefore, the research approval by an Institutional Review Board (IRB) was not required.

### Data

The analysis draws on observational data for 50 African countries over the period 1990–2017 at the country level. Data on conflicts stem from two different data sets. The first data set relates to small and medium-sized incidents of social violence ranging over a variety of different event types (protests, demonstrations, riots, strikes, and other forms of social disturbances). The respective information is obtained from the Social Conflict Analysis Database (SCAD) – a compilation based on global press coverage available for the period 1990–2017. These data also contain information on organized vs. spontaneous events, on specific underlying motivations (events related to elections, economic grievances, or human rights), and on event sizes (in terms of casualties and participants), and actors involved. While offering a comprehensive data set on social conflict events, SCAD is based on two main sources, Associated Press (AP) and Agence France Presse (AFP). We therefore replicate the analysis with an alternative data set.

The second, complementary, data set provided by the Uppsala Conflict Data Program (UCDP) covers large-scale civil conflicts. These data, which we refer to as armed conflict, include information about events that involve organized violence of substantial intensity. They include organized large-scale conflict events involving the state, and contain information on geo-localization, date, type and actors involved in a given event.

Data on HIV prevalence and ART coverage at the country level are provided by UNAIDS for 50 African countries. HIV prevalence information is based on model estimates that are comparable across regions and countries over time. Data on ART coverage is based on national registers. Information on global prices of the first line of combined ART treatment, which is used later on, is from WHO Global Price Reporting Mechanism and by the Global Fund Pooled Procurement Mechanism Reference Pricing. These data measure the costs of the active pharmaceutical ingredients used in the production of the treatment lines and covering approximately 70–80 percent of the global transactions of the respective drugs. Data on population and life expectancy at birth is from the UN Population Division and various Census reports. Detailed information about all data sources, the variable definitions and data construction is contained in the supporting information (S1 Text). Table A1 in S1 Text contains summary statistics for the variables of main interest.

### Methods

We re-investigate the link between health improvements, population dynamics, and conflict, based on the conceptual framework illustrated in Fig 1. To isolate the causal effect of population on conflict, we use an identification strategy based on instrumental variables. Specifically, we instrument population (the endogenous variable) using exogenous variation from one of the largest health campaigns of the last two decades in Africa: the massive roll-out of antiretroviral therapy (ART) in the context of the global fight against the human immunodeficiency virus (HIV). The instrumental variable approach exploits variation in the availability of antiretroviral therapy (ART) to HIV-positive individuals during the early 2000s that had an effect on population dynamics, but that is plausibly exogenous to conflict in a particular country. The background of this identification strategy is as follows. HIV impairs the functioning of CD4 (white blood) cells in the immune system, making patients vulnerable to infections and other diseases such as cancer. In advanced stages, the infection turns into the acquired immune deficiency syndrome (AIDS), which leads to death if untreated. Emanating from Kinshasa around 1920 [15], HIV swept across Africa and beyond from the late 1970s onwards. By 2000, an estimated 26 million people lived with HIV/AIDS in Africa, which corresponded to more than 70 percent of global infections (see, e.g., reports by UNAIDS/WHO). As result of intense research activities, the retrovirus responsible for AIDS was first isolated in 1983. The subsequent identification of the main receptors of the HIV paved the way for the development of combination antiretroviral therapy (ART) in the late 1990s [16]. Mounting pressures from non-government organizations led to the introduction of generic ART drugs in 2001 that lowered the prices for ART drugs and made them more affordable. The subsequent rapid expansion of ART availability and access to treatment throughout the world supported by the Global Fund transformed HIV infections from a fatal infection to a manageable chronic disease with moderate implications for life expectancy if treated appropriately. This health intervention prevented an estimated 9.5 million deaths and brought considerable economic benefits [17], mainly by contributing to a substantial reduction in mortality [18,19] and leading to a recovery of labor productivity [20,21].

To investigate the empirical relevance of step I. of the conceptual framework, the analysis applies an estimation framework that is based on a differences-in-differences logic. The first of the two corresponding contrasts reflects cross-sectional heterogeneity and stipulates that the global ART expansion is expected to have more pronounced effects on population in countries that exhibit a larger scope for health improvement and mortality decline. This cross-sectional scope for ART, denoted by *Z*_*c,*2001_, is measured by the country-specific prevalence of HIV infections in 2001, just before ART became widely available. The second of the two contrasts reflects the dynamics in access to ART. This variation over time, denoted by *ART*_*IV,t*_, is measured by global ART coverage outside Africa, or, alternatively, by the price for ART treatment or the production cost for ART drugs.

The estimation combines these two dimensions, the potential for ART, *Z*_*c,*2001_, and the global variation over time in the access to ART treatment responsible for the ART expansion, *ART*_*IV,t*_, to construct a measure of the country-specific expansion of ART coverage that allows isolating the effect of the health intervention on population. The inclusion of country fixed effects (i.e., country-specific constant terms) and control variables accounts for systematic variation across countries, and the inclusion of time fixed effects (i.e., separate dummy variables for each year) accounts for factors common to all countries. In addition, the analysis accounts for time trends for macro regions within Africa, and of country-specific trends related to HIV prevalence.

From the perspective of the conceptual framework illustrated in Fig 1, the global roll-out of ART constitutes an ideal natural experiment to investigate the impact of variation in population on conflict. In particular, it allows combining cross-sectional variation in the ART treatment potential (drawing on HIV prevalence before the actual availability of treatment at the country level) with variation in ART access over time at the global level (exploiting global variation over time in ART coverage as well as in production costs and prices of ART). This combination of pre-determined cross-sectional treatment potential and time variation in ART access, both of which are unrelated to subsequent conflict outcomes, satisfies the exclusion restriction by affecting conflict only through its effect on population growth.

The rationale for the instrumentation approach is based on the same differences-in-differences logic as described before. Besides being a relevant predictor of ART coverage (as documented above), the instrument must satisfy the exclusion restriction (the instrument should affect conflict only through its effect on population). We combine the potential for ART, *Z*_*c,*2001_, and global variation over time in the access to ART treatment responsible for the ART expansion, *ART*_*IV,t*_, to predict the effect on population growth of the expansion in ART coverage. This approach makes use of variation over time and across countries that is plausibly exogenous to local conflict events and hence satisfies the exclusion restriction for an instrument. Specifically, the variation over time makes use of the global expansion of ART availability that occurred for reasons unrelated to conflict-related events within a particular African country. The variation across countries reflects the country-specific scope for health improvements due to the ART expansion and is plausibly exogenous conditional on the inclusion of country fixed effects and control variables that account for systematic variation in conflict activity across countries (e.g., sea access, the prevalence of natural resources, or mountainous terrain may influence the risk of conflict). Moreover, the inclusion of time fixed effects accounts for factors that influence conflict in a given year and that are common to all countries (such as global price fluctuations for energy or food commodities, pandemics, or the war on terrorism) and the inclusion of time trends for macro regions within Africa, and of country-specific trends related to HIV prevalence accounts for trends in specific regions (e.g., the increase in Islamic militant violence in Northern Africa during the early 2000s).

Conceptually the exclusion restriction is analogous to that in previous research that found that population growth fuels conflict [11]. Yet, it requires less stringent assumptions to be satisfied. Specifically, in comparison to the context of a health intervention that addresses several diseases and whose effects on conflict are measured over more than half a century, the expansion of antiretroviral therapy only reduced mortality from HIV over a relatively short and concentrated period of time.

Besides addressing the research question using contemporaneous data, our approach represents a step forward methodologically. Compared to earlier work, our analysis isolates the effect of a well-defined single health intervention related to a single, specific disease and that was effective within a comparably short period of time, rather than exploiting variation from various interventions related to several diseases over nearly half a century. Instead of using a proxy for the latent mortality decline that is based exclusively on the cross-sectional pre-treatment variation in mortality, our approach draws on cross-sectional variation in treatment scope in combination with measures of time variation in treatment intensity that is based on the actual treatment intensity outside Africa, or, alternatively, on global dynamics of costs and prices of drugs. In combination with high resolution data, our analysis also addresses a series of methodological challenges that have plagued the existing literature, such as measurement error, unobserved heterogeneity and potential confounders, by accounting for time-invariant confounders and global shocks. Earlier literature on the global roll-out of ART has documented a positive effect on life expectancy and economic growth, but without linking it to conflict [18,19]. Previous research estimated the effect of health interventions on conflict in reduced form while abstracting from the role of population dynamics for conflicts [22]. By adopting the conceptual framework illustrated in Fig 1 and providing novel evidence on the impact of health driven population growth on conflict outcomes, our analysis offers the first attempt to “connect all the dots” and explore the chain of mechanisms.

## Results

Our analysis documents the population dynamics resulting from the global roll-out of ART and then investigates their effect on violent conflicts in Africa over the period 1990–2017. The unit of observation is a country-year observation.

### Stage I. The impact of ART on population growth

The empirical evidence for Stage I. of the conceptual framework depicted in Fig 1 documents that the global ART expansion, as reflected by the interaction between cross-country variation in the scope for ART expansion with global variation in the access to ART over time, led to a significant acceleration of population growth (Table 1). This does not only hold in reduced form, but also when applying an instrumental variable approach to predict ART prevalence in a country (Table A2 in S1 Text). Quantitatively, the results imply that one standard deviation increase in ART coverage of HIV infected individuals (equivalent to 18.6%) is associated with an increase in population growth of 0.15-0.2 standard deviations across different reduced form specifications, and of 0.95 standard deviations in instrumental variables estimates, which corresponds to approximately 1% population growth (see Tables A1 and A2 in S1 Text). These results confirm the earlier findings that successful health interventions lead to accelerated population increases, as stipulated by the Malthusian mechanism. They also provide an indication for the usefulness of the instrumentation strategy. Specifically, simple regressions of population on ART coverage might suffer from measurement error in ART coverage or omitted variables which tend to reduce population growth (e.g., if greater ART coverage is also associated to better availability of contraception), with the result of the coefficient estimate being biased towards zero. Using an instrument based on the interaction of the country-specific scope for ART and the global variation over time in the access to ART treatment responsible for the ART expansion helps reducing measurement error and avoids bias from omitted variables in the coefficient of interest.

**Table 1:** Stage I: Effect of ART expansion on population growth.

Growth of Population (log)			
	(1)	(2)	(3)
^*Z*^_*i,*2001_ ^*× ART*^^*IV,t*^	0.212*** (0.065)	0.162*** (0.046)	0.154*** (0.043)
^*Z*^_*i,*2001_	*HIV* 2001_*c,*2001_	*HIV* 2001_*c,*2001_	*HIV* 2001_*c,*2001_
*ART*_*IV,t*_	ART Cov	ART Price	ART Cost
Observations	1,344	1,344	1,344
Clusters	50	50	50
R-squared	0.06	0.06	0.06
*Country f.e.*	Yes	Yes	Yes
*Year f.e.*	Yes	Yes	Yes
*Region Time Trend*	Yes	Yes	Yes
*HIV Trend*	Yes	Yes	Yes

*Note:* The table presents results for Stage I. of the conceptual framework of Fig 1, document- ing the impact of ART (Antiretroviral Therapy) expansion on population growth. Estimation results refer to estimates of the coefficient *α* from an empirical model:

$\Delta {\text{Population}}_{c,t}=\alpha \cdot {ART}_{c,t}+{\gamma X}_{c,t}+\delta_{c}+\zeta_{t}+\rho_{R}\cdot t+u_{c,t}$

with as dependent variable the growth rate of population (measured as ln(population_*t*_) − ln(population_*t−*1_)) at the country level, the measure is standardized. Data source: World Bank. ART: ART coverage based on data from UNAIDS, measure standardized. OLS estimates from an empirical model with *ART*_*c,t*_ being measured by *Z*_*i,*2001_ *× ART*_*IV,t*_, which combines the cross-sectional variation in the local scope for ART at the country level, *Z*_*c,*2001_, and the global variation over time in the access to ART, *ART*_*IV,t*_; the interaction term has been standardized (beta coefficient). All coefficients refer to standardized explanatory variables. Results for time period 1990–2017. All empirical specifications include country fixed effects *δ*_*c*_, year fixed effects *ζ*_*t*_, African-subregion-specific linear time trends *ρ*_*R*_ *·t*, and as control variables *X_c,t_* a linear time trend interacted with HIV prevalence in a country in 2001.

*/**/*** indicate significance at 10%/5%/1%, respectively; standard errors in parentheses clustered at the country level.

### Stage II. The impact of health-driven population growth on conflicts

Evidence for Stage II. of the conceptual framework depicted in Fig 1 for developing countries today is still lacking. Here we provide such evidence focussing on two types of conflict: social violence in terms of riots or protests, and large-scale armed conflict that involves organized violence of substantial intensity. To address the question whether the acceleration of population growth induced by the global ART expansion also led to more social conflict, the analysis applies a two-stage estimation approach that uses the same combination of cross-sectional variation in the local scope for ART expansion and global variation over time in the access to ART as instrumental variable on the first stage. This empirical strategy allows estimating the causal effect of population growth on conflicts by accounting for the fact that population may be endogenous to violence or to third factors that affect both violence and population.

**Fig 2:**
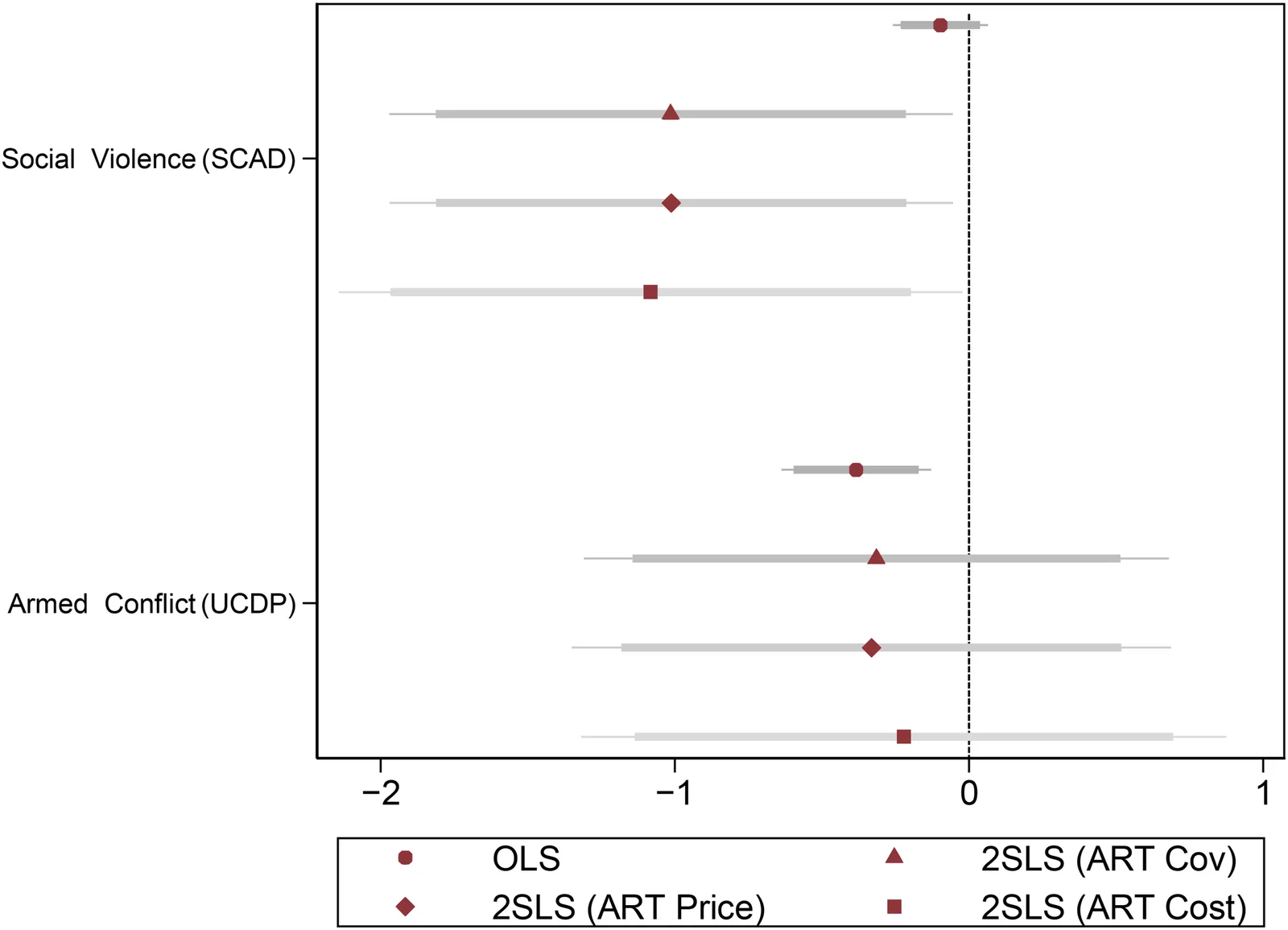
Stage II: Effect of ART-driven population growth on conflict.

Fig 2 presents the empirical results in graphical form. The corresponding regression results are contained in Table A4 in S1 Text. The first panel of Fig 2 focuses on social violence in the form of small and medium-sized incidents of conflict, relying on SCAD data. OLS regression results of conflict on population growth (first bar) show a non-significant effect. The results for the instrumental variables approach, which are based on the causal identification strategy discussed above and implemented in a two-stage least squares (2SLS) estimation framework, document that population growth due to the ART expansion led to a statistically significant *decrease* in social violence. Quantitatively, an increase in population growth of 10% of a standard deviation entails a 6.6% reduction in the number of events of social violence. When considering ongoing armed conflicts, as measured by UCDP data in the bottom panel, we also find negative coefficient estimates. The effect is negative and significant in the OLS estimates, but we do not detect a statistically significant impact of population growth on armed conflict in the instrumental variable estimations.

The validity of the instrumental variables estimates requires the exclusion restriction that the instrument affects conflict only through its effect on population to be satisfied. This exclusion restriction is plausibly satisfied in the present context because, conditional on controls and fixed effects, there are no credible alternative direct channels linking the instrument to violence. Potential threats to this exclusion restriction include direct effects of HIV reduction on income, education or trust.

Additional results support the validity of the exclusion restriction by showing that the coefficients remain qualitatively unaffected when additional controls are added. In the SI, we document that our results also hold when explicitly controlling for these covariates (income, education, and trust, see Tables A4 and A5 in S1 Text), or when controlling for institutional quality or overall aid disbursements (Tables A6 and A7 in S1 Text).

## Discussion

This paper reconsiders the question of whether population expansions induced by health interventions and the related decline in mortality cause a surge in conflict. We empirically investigate this question by looking at one of the largest health interventions in developing countries in the recent past: the global expansion of antiretroviral therapy (ART). We follow a similar conceptual and empirical approach as recent literature that has reported evidence in favor of the conjecture of Malthusian conflict dynamics, yet we are able to draw on a novel, very well-defined health intervention and fine-grained data. The results indicate that the population growth spurred by the successful roll-out of anti-retroviral therapy and the resulting mortality decline was not a culprit for conflict. This contradicts earlier work that pointed at a rise in conflict as a potential negative consequence of health interventions in the past [11]. Instead, the results rather align with findings of conflict reduction as the result of successful health prevention or health interventions [22,23]. From a policy perspective, this converging evidence suggests that concerns about health interventions causing more conflict through their effects on population might be misplaced. Complementing earlier evidence for the positive direct effects of the ART expansion on health and labor market outcomes, our findings suggest that health campaigns do not lead to more conflict but instead might even help to alleviate conflict, thereby providing a second “dividend” beyond improving health.

The empirical findings refer to the effects of successful health interventions that were the result of international efforts. The evidence presented here suggests that Malthusian conflict dynamics in response to health improvements might be less of a concern than thought previously. The results and the conceptual framework suggest some important directions for future research and the further exploration of the underlying mechanisms. Existing work on long-run growth has pointed at the importance of the demographic transition and the reversal in the relation between economic living conditions and population growth that is typically associated with greater investment in child health and education [24]. Evidence based on variation from the epidemiological transition, that conceptually resembles the empirical approach of this paper, has found that health improvements after the demographic transition lead to a moderation in population growth and thus an acceleration of economic development through fostering education and labor force participation [25,26]. This suggests that, by increasing the value of human capital and the expected future returns from productive activities, better health and longer life expectancy make violence a less attractive choice by increasing the opportunity costs for conflict [27]. Consistent with this view, work on the HIV/AIDS epidemic suggests that the HIV-related increase in mortality led to a decline in human capital investment [28,29], whereas the expansion of antiretroviral therapy seems to have stopped this development and lead to an economic recovery [19].

Moreover, in most African countries, the demographic transition is on its way, raising questions about the magnitude of Malthusian conflict dynamics in the future. Nevertheless, another lesson of the results for governments and organizations is that policies that accommodate conflict-intensifying consequences of population dynamics, such as intensified resource scarcity, might be desirable to help avoiding that health interventions cause Malthusian conflict dynamics.

Naturally, the present analysis has limitations that need to be kept in mind when interpreting the results. The evidence documents that the population increases caused by the global ART expansion did not cause more conflict on the African continent.

Whether this finding extends to the population dynamics caused by health interventions targeting other diseases, or on other continents or during other time periods is an empirical question. Furthermore, while the analysis isolates the causal effect of population dynamics on conflict, more work is needed to isolate the precise channels and mechanisms through which the effect of population on conflict operates.

## Conclusion

The empirical evidence presented here does not support the conjecture that health-driven population growth fuels social violence and armed conflict. On the contrary, the findings suggest that the population expansion induced by the successful roll-out of antiretroviral therapy (ART) for HIV-positive individuals during the early 2000s entailed no effect on armed conflict and even reduced social violence. Overall, the results do not point at the existence of a relevant trade-off for health interventions between mortality decline and conflict increase, particularly for today’s poorest, conflict-prone countries. The policy implication following from this result is that investments in health improvements might yield a “double dividend”: They improve health conditions but might even reduce conflict. The results suggest avenues for further research for a better understanding of the consequences of other health interventions and of the specific mechanisms linking health improvements, population dynamics, and conflicts.

## Supporting information

S1 Text The supporting materials include the following information: Supporting data, additional tables. (PDF)
