# Supplementary material for: Health interventions do not cause Malthusian conflict dynamics: Evidence from the expansion of HIV treatment in Africa
Source: PLOS Glob Public Health. 2026 Aug 19;6(8):e0005448. doi: 10.1371/journal.pgph.0005448 (PMC13489451; doi:10.1371/journal.pgph.0005448)
Supplement: Supplementary file 1 [file pgph.0005448.s001.pdf]

Supporting Materials

Health interventions do not cause Malthusian conflict dynamics: Evidence from the expansion of HIV treatment in Africa

Andrea Berlanda[1], Matteo Cervellati[2,3], Elena Esposito[3,4],
Dominic Rohner[3,5,6,*], Uwe Sunde[3,7]

1 Department of Economics, University of Bergamo, Bergamo, Italy
2 Department of Economics, University of Bologna, Bologna, Italy
3 CEPR, London, UK
4 ESOMAS Department, University of Torino, Torino, Italy
5 Department of Economics, Geneva Graduate Institute, Geneva, Switzerland
6 Department of Economics, University of Lausanne, Lausanne, Switzerland
7 Department of Economics, University of Munich (LMU), Munich, Germany
* dominic.rohner@unil.ch.

Content

All data required to produce the results presented in the paper and in the Supporting Materials are publicly accessible at Harvard Dataverse (doi:10.7910/DVN/BPHYM8). This document contains the following Supporting Materials:

- Supporting Data
  - Data: Conflict (SCAD, UCDP)
  - Data: HIV Prevalence
  - Data: Antiretroviral Therapy (ART) Coverage
  - Data: Antiretroviral Therapy (ART) Costs and Prices
  - Data: Other Variables and Sources
- Additional Tables
  - Table A1 shows summary statistics for the main variables of the analysis.
  - Table A2 shows that ART does not only prediction population growth in reduced form, but also when following an instrumental variable approach.
  - Table A3 contains the estimation results underlying Figure 2 of the main text. This table contains additional information on the number of observations and other technical details of the specifications used.
  - Table A4 reproduces the main results for an extended empirical model that additionally controls for income and education

- Table A5 reproduces the main results for an extended empirical model that additionally controls for trust. Given that the trust data contains missing observations, the estimates partly rely on imputed data (see table note). The results with a control for trust are presented in a separate table to prevent a further drop in observations. The results of Table A5 should be interpreted with caution.
- Table A6 reproduces the main results for an extended empirical model that additionally controls for institutional quality and democracy scores.
- Table A7 reproduces the main results for an extended empirical model that additionally controls for aid disbursements of the Global Fund.

Supporting Data

Data: Conflict

Social Conflict Analysis Database - SCAD

Primary Sources and Data Construction. Data on events of social violence is based on information from the Social Conflict Analysis Database –SCAD [*1*]. These data contain information on protests, demonstrations, riots, strikes, and other forms of social disturbances in Africa. The data set does not include violent events related to organized armed conflict such as rebellions, civil wars, and international war. The SCAD data and documentation about coding procedures can be accessed at `https://www.strausscenter.org/scad.html` (last accessed 20 October 2023).

The SCAD database covers all countries with a population of more than 1 million in Africa and compiles events reported by Associated Press (AP) and Agence France Presse (AFP), accessed through Lexis-Nexis. The relevant articles have been selected according to a search protocol based upon keyword searches of country names and five terms: "protest," "riot," "strike," "violence," and "attack". Before coding, each retrieved article was then examined by a member of the SCAD team, discarded if not related, and otherwise kept and coded into different types of events. The SCAD team double-coded 10 percent of the country-years to verify the accuracy of the coding procedures. Particular attention was devoted to avoid double or triple counting a single event, whenever an event was documented by multiple articles. Last, the geographic location of the event was added by searches on a wide variety of platforms.

Construction of Data for Analysis. Over the period 1990-2017, 17,644 events, localized across 50 African countries, are retrieved. The only event category excluded is Intra-Government violence, which represents events reflecting social conflict within the army or the police forces. Events are linked to the respective country/region based on information about latitude and longitude. To generate a measure of social violence at sub-national levels, only geo-localized events are used. The main measure of social violence denotes the total number of violent events in the country/region in a given year.

The data set used in the analysis covers 50 African countries over the period 1990-2017. The number of events ranges between a minimum of 0 to a maximum of 617, with an average of 11.9 events per country/year. More than 77% of all country-year observations exhibit at least one event. For a subset of events (7,750 out of 17,644 events) information on the approximate number of participants of the event is available.

Uppsala Conflict Data Program (UCDP)

Primary Sources and Data Construction. Data on conflict events are based on on information collected by the Uppsala Conflict Data Program (UCDP GED). The UCDP data contain violent events, defined as "the incidence of the use of armed force by an organized actor against another organized actor, or against civilians, resulting in at least 1 direct death in either the best, low or high estimate categories at a specific location and for a specific temporal duration [*2*]. The data contain information about location, timing, and involved actors of events.

UCDP data are based on automated searches of global news companies, media, international organizations, NGOs, historical achives and other sources of information, which are then evaluated by human coders. UCDP data and documentation can be accessed at `https://www.pcr.uu.se/research/ucdp/`.

Definitions of Event Types. UCDP data contain information on armed conflicts, defined as a contested incompatibility that concerns government and/or territory where the use of armed force between two parties, of which at least one is the government of a state. UCDP data only contain organized events involving at least one fatality; incidents without information on fatalities are not included. Conflict events in the UCDP data that meet these criteria therefore represent organized violence of considerable intensity.

Construction of Data for Analysis. Over the period 1990-2017, 35,264 events, localized across 50 African countries, are retrieved. Events are linked to the respective country. The number of events ranges between a minimum of 1 to a maximum of 583, with an average of 55.1 events per country/year. All of the country-year observations in Africa exhibit at least one event.

Comparison of Conflict Data from SCAD and UCDP

The SCAD data focus on social violence defined as social and political unrest, excluding large-scale organized armed conflicts. The data has long time coverage and high data quality. In particular, the procedures of data construction of the SCAD data avoid duplication of events, which is a particularly serious concern for comparable data sets – especially for data sets that (unlike SCAD) rely exclusively on automated text extraction algorithms. The same sources are used for retrieving episodes of violence for all countries/regions/years in the sample – which ensures a consistent coding.

The UCDP data represent information about social conflict that involves organized violence of substantial intensity. This measure complements the SCAD data, which excludes all events defined as "a contested incompatibility that concerns government and/or territory where the use of armed force between two parties, of which at least one is the government of a state, results in at least 25 battle-related deaths in one calendar year". This makes it an ideal complementary source of information to investigate the results for different types of civil conflict.

Data: HIV Prevalence

At the country level, data for HIV prevalence are obtained from UNAIDS (downloaded from the World Bank data platform `https://data.worldbank.org/indicator/SH.DYN.AIDS.ZS`). Since the exact number of individuals living with HIV cannot be determined in many countries, the best possible approximation is obtained through model estimates. For each country, UNAIDS composes teams of experts (epidemiologists, demographers, monitoring and evaluation specialists and technical partners) who collect and process data to produce estimates for HIV prevalence. UNAIDS collects all country estimates and reviews them through the work of the Strategic Information and Monitoring Division, in order to guarantee that estimates are comparable across countries over time. The construction of estimates differs across countries with high and low levels of HIV prevalence:

- For *high-level HIV epidemic countries* the estimates are based on data from surveillance among pregnant women and from nationally representative population-based surveys.
- For *low-level HIV epidemic countries* the estimates are based on data from sub-populations at high risk of HIV infections that are combined with nationally representative surveys to produce the estimates.

Construction of Data for Analysis The analysis is based on HIV prevalence among the population group with age 15-49 years. Formally, this variable is defined as HIV Prevalence = number HIV infected (15-49 years) / total population (15-49 years). The data set covers 50 African countries over the period 1990-2017. The average HIV Prevalence (15-49) is 0.047, ranging from a minimum of 0.001 to a maximum of 0.285 (Swaziland in 2013 and 2014).

Data: Antiretroviral Therapy (ART) Coverage

Data for coverage with antiretroviral (ARV) therapy at the country level is obtained from UNAIDS (downloaded from the World Bank data platform `https://data.worldbank.org/indicator/SH.HIV.ARTC.ZS`). ART coverage indicates the percentage of all individuals living with HIV who receive antiretroviral therapy. In each country, local facilities administering antiretroviral therapy hold registers that are compiled and sent to national authorities on a routine basis. UNAIDS requests countries to submit these data on the 31st of March every year through an on-line reporting tool. The tool features several quality checks in order to avoid reporting errors; UNAIDS further validates the data, comparing them with information from alternative sources.[1]

Construction of Data for Analysis (ARV Cov). The analysis makes use of ART coverage measured as the share of treated individuals relative to the total population in a country. Formally, this variable is defined as ARV Coverage (percentage population) = number ARV treated / total population. As alternative measure for the robustness analysis, the results are cross-validated using the number of treated individuals relative to the number of HIV infected persons. Formally, this variable is defined as ARV Coverage (out of Infected Individuals) = number ARV treated / number HIV-infected. The data set covers 50 African countries over the period 1990-2017. The average ARV Coverage (out of Infected Individuals) is 0.12, ranging from a minimum of 0 to a maximum of 0.85 (Swaziland in 2017). The identification strategy makes use of data for 53 low and middle income countries outside Africa for which ART coverage is available.

Data: Antiretroviral Therapy (ART) Costs and Prices

Data on the time-series of ART Prices and Production Costs are based on the combination of information from two different sources: The WHO Global Price Reporting Mechanism (GPRM) and the Global Fund Pooled Procurement Mechanism Reference Pricing. The WHO Global Price Reporting Mechanism (GPRM) was created in 2003 to support transparency in the market of antiretroviral (ARV) drugs. Until 2013, GPRM reported prices and volumes of ARV drugs sold on international markets covering 132 countries (35 low-income countries, 44 lower-middle income, 36 upper-middle income and 17 high income countries). The main providers of data were the Global Fund, PEPFAR, UNITAID, and the procurement organizations working with them, such as the Clinton Foundation, Crown Agent, the Global Drug Facility (GDF), the International Dispensary Association (IDA HIV/AIDS), USAID/Deliver, Mission Pharma, Management Sciences for Health (MSH), the Partnership for Supply Chain Management (PFSCMS), the United Nations Development Programme (UNDP), the United Nations Children's Fund (UNICEF), and the WHO/Contracting and Procurement Service (WHO/CPS). From 2004 to 2012 the GPRM covered

[1] Further information can be obtained from: `https://www.unaids.org/en/dataanalysis/knowyourresponse/HIVdata_estimates`.

between 70 and 80 percent of total volume of ARV drug transactions [3]. The analysis here makes use of price data for active pharmaceutical ingredient (API) from GRPM. These data contain the median price for the most relevant treatment regimens and their API over the period 2003-2012. Since 2015, the Global Fund Pooled Procurement Mechanism Reference Pricing has published quarterly reports containing reference prices for ARV medicines. Reports from the Global Fund were retrieved from `https://www.theglobalfund.org/en/sourcing-management/health-products/antiretrovirals/andhttps://www.who.int/hiv/amds/gprm/en/`.

Construction of Data for Analysis – Prices (ART Price). The main analysis focuses on the median price of a specific and widely used first line treatment for adults, ZDV-3TC-EFV, which represents a combination of three reverse-transcriptase inhibitors: 300mg of Zidovudine (ZDV), 150mg of Lamivudine (3TC) and 600 mg of Efavirenz (EFV).[2] Since data are available for the period between 2003 and 2012 and from 2015 to 2017, data for the years 2013 and 2014 have been linearly interpolated. The prices for the ZDV-3TC-EFV first line treatment ranges from 644$ US per patient per year in 2003 to 102$ US in 2017. For comparability with the results based on ART coverage in non-African countries, prices are converted into a reverse index.[3]

Construction of Data for Analysis – Costs (ART Cost). The production costs of the same first line treatment ZDV-3TC-EFV are available only for selected years, namely 2005, 2007, 2010 and 2012. The time-series of production cost were constructed as follows. First, exploiting prices and costs for available years, we computed the mark-up of pharmaceutical companies (ARV medicine prices/API costs). Second, this mark-up is assumed to be constant before 2005 and after 2012, when cost information is not available; in this way it is possible to compute, in a very conservative way, costs over the periods 2003-2005 and 2013-2017 by combining the predicted mark-ups with the complete time-series of price data. Third, for the missing years between 2005 and 2012, we interpolated the data to build a continuous estimate of the production costs. The API production cost for ZDV-3TC-EFV ranges from a maximum of 477$ in 2003 to a minimum of 86$ per patient per year in 2017. For comparability with the results based on ART coverage in non-African countries, costs are converted into a reverse index.[4]

Data: Other Variables and Sources

- **Time Trends for African Regions**: The main specification accounts for differential trends in different regions of Africa, based on the classification of subregions according to the United Nations geoscheme for Africa. Countries are coded as follows: *Central Africa:* Burundi, Cameroon, Central African Republic, Chad, Congo, Dem. Rep. Congo, Rep. Equatorial Guinea, Gabon; *East Africa*: Comoros, Djibouti, Eritrea, Ethiopia, Kenya, Madagascar, Rwanda, Somalia, South Sudan, Sudan, Tanzania, Uganda; *North Africa*: Algeria, Egypt, Arab Rep. Mauritania, Morocco, Tunisia; *Southern Africa*: Angola, Botswana, Eswatini (Swaziland), Lesotho, Malawi, Mozambique, Namibia, South Africa,

[2]The analysis focuses on first line regimens for adults, which represent the most widely used treatments of the time and were substantially less expensive than second line treatments. This particular first line regimen is considered the most effective regimen and is the one recommended by WHO. Furthermore, because of its higher initial price it was initially not widely used in low and middle income countries. Alternative regimens (in particular the second first line regimen ZDV-3TC-NVP) are used for robustness checks on the instrumentation.

[3]In particular, the price index for year t is constructed as $price\_index_t = (price_{2003}/price_t) - 1$.

[4]In particular, the cost index for year t is constructed as $cost\_index_t = (cost_{2003}/cost_t) - 1$.

Zambia, Zimbabwe; *West Africa*: Benin, Burkina Faso, Cabo Verde, Cote d'Ivoire, Gambia, Ghana, Guinea, Guinea-Bissau, Liberia, Mali, Niger, Nigeria, Senegal, Sierra Leone, Togo.

- **Population**: Population data are taken from United Nations Population Division, Census reports, Eurostat, United Nations Statistical Division, U.S. Census Bureau and Secretariat of the Pacific Community and downloaded from the World Bank data platform (`https://data.worldbank.org/indicator/SP.POP.TOTL`). Total population is based on the de facto definition of population, which counts all residents regardless of legal status or citizenship. The values represent mid-year estimates.
- **Education**: Taken from UNESCO Institute for Statistics (UIS) and downloaded from the World Bank data platform (`https://data.worldbank.org/indicator/se.sec.enrrandhttps://data.worldbank.org/indicator/SE.PRM.ENRR`). We measure education as primary school enrollment or secondary school enrollment.
- **Trust**: Trust data comes from Afrobarometer (round 1 to 6) covering the period from 1999 to 2015. We include information for all the available countries and interpolated observations for the missing years to obtain measures of the shares of individuals in each country who trust the president, the parliament, and the ruling party.

Additional Tables

Table A1. Summary Statistics

	N	Mean	Min.	Max.	Std.Dev.
HIV prevalence (15-49) in 2001	1344	0.056	0.001	0.268	0.070
ART treatment	1344	0.121	0.000	0.850	0.186
Population Growth	1344	0.025	-0.062	0.079	0.010
GDP growth	1251	0.015	-0.648	0.877	0.063
UCDP events (total)	1344	24.657	0.000	583.000	66.260
Social Violence, SCAD	1344	12.209	0.000	617.000	31.783

Note: Summary statistics at the level of countries for the period 1990-2017. HIV prevalence as percentage of the population aged 15-49 in the year 2001.

Table A2. Effect of ART Expansion on Population Growth

	Growth of Population (log)			
	OLS	2SLS		
	(1)	(2)	(3)	(4)
ART	0.116	0.945***	0.968***	0.957***
	(0.086)	(0.329)	(0.312)	(0.312)
$Z_{i,2001}$		$HIV2001_{c,2001}$	$HIV2001_{c,2001}$	$HIV2001_{c,2001}$
$ART_{IV,t}$		ART Cov	ART Price	ART Cost
Observations	1,344	1,344	1,344	1,344
Clusters	50	50	50	50
Kleibergen-Paap		32.41	31.92	29.45
Country f.e.	√	√	√	√
Year f.e.	√	√	√	√
Region Time Trend	√	√	√	√
HIV Trend	√	√	√	√

Note: The table presents results documenting the impact of ART (Antiretroviral Therapy) expansion on population growth using OLS, 2SLS, and Reduced Form Estimates. Estimation results from an empirical model:

$$\Delta\text{Population}_{c,t} = \alpha \cdot ART_{c,t} + \gamma X_{c,t} + \delta_c + \zeta_t + \rho_R \cdot t + u_{c,t}$$

with dependent variable growth rate of population (measured as $\ln(\text{population}_t) - \ln(\text{population}_{t-1})$) at country level, the measure is standardized. Data source: World Bank. ART: ART coverage based on data from UNAIDS, measure standardized. Column 1: OLS estimates. Columns 2-4: 2SLS estimates of the effect on violent events of instrumented ART coverage; in 2SLS estimates, ART coverage is instrumented using $Z_{c,2001} \cdot ART_{IV,t}$, which combines the cross-sectional variation in the local scope for ART at the country level, $Z_{c,2001}$, and the global time-series variation in the access to ART, $ART_{IV,t}$; the interaction term has been standardized; see text for details. All coefficients refer to standardized explanatory variables. Results for time period 1990–2017. All empirical specifications include country fixed effects δ_c, year fixed effects ζ_t, African-subregion-specific linear time trends $\rho_R \cdot t$, and as control variables $X_{c,t}$ a linear time trend interacted with HIV prevalence in a country in 2001. */**/*** indicate significance at 10%/5%/1%, respectively; standard errors in parentheses clustered at the country level.

Table A3. Effect of Population Growth on Conflicts

	Social Violence (log events) - SCAD Data			
	OLS	2SLS		
	(1)	(2)	(3)	(4)
Std Growth of Population (log)	-0.097	-1.082**	-1.014**	-1.012**
	(0.080)	(0.527)	(0.477)	(0.477)
$Z_{i,2001}$		$HIV2001_{c,2001}$	$HIV2001_{c,2001}$	$HIV2001_{c,2001}$
$ART_{IV,t}$		ART Cov	ART Price	ART Cost
Observations	1,344	1,344	1,344	1,344
Clusters	50	50	50	50
Kleibergen-Paap		10.65	12.66	12.54
	**Ongoing Armed Conflicts (log events) - UCDP Data**			
	OLS	2SLS		
	(1)	(2)	(3)	(4)
Std Growth of Population (log)	-0.383***	-0.221	-0.314	-0.331
	(0.127)	(0.546)	(0.495)	(0.507)
$Z_{i,2001}$		$HIV2001_{c,2001}$	$HIV2001_{c,2001}$	$HIV2001_{c,2001}$
$ART_{IV,t}$		ART Cov	ART Price	ART Cost
Observations	1,344	1,344	1,344	1,344
Clusters	50	50	50	50
Adj-R2	0.04	0.03	0.04	0.04
Kleibergen-Paap		10.65	12.66	12.54
Country f.e.	√	√	√	√
Year f.e.	√	√	√	√
Region Time Trend	√	√	√	√
HIV Trend	√	√	√	√

Note: OLS and 2SLS estimates of β obtained from the model

$$\text{Conflict}_{c,t} = \beta \cdot \Delta\text{Population}_{c,t} + \gamma X_{c,t} + \delta_c + \zeta_t + \rho_R \cdot t + \varepsilon_{c,t}$$

The dependent variable is social conflict in country c and year t, measured as log events of conflict from the different data sets (SCAD and UCDP). The explanatory variable of interest corresponds to growth of population in country c and year t, instrumented using $Z_{c,2001} \times ART_{IV,t}$. Instrument: interaction between cross-sectional variation in the potential for ART treatment, $Z_{c,2001}$ (measured by HIV prevalence at country level, 2001), and a time-varying measure of ART expansion, $ART_{IV,t}$ (measured by global ART coverage outside Africa, or, alternatively by the global variation in the median world price of ART treatment regimens, ART Price, or by the cost of ART treatment regimens, ART Cost); the interaction term has been standardized. All empirical specifications include country fixed effects δ_c, year fixed effects ζ_t, African-subregion-specific linear time trends $\rho_R \cdot t$, and as control variables $X_{c,t}$ a linear time trend interacted with HIV prevalence in a country in 2001. All coefficients refer to standardized explanatory variables.

Table A4. Robustness: Effect of Population Growth on Conflicts controlling for Income and Education

	Social Violence (log events) - SCAD Data			
	OLS	2SLS		
	(1)	(2)	(3)	(4)
Std Growth of Population (log)	-0.125***	-1.572**	-1.393**	-1.370**
	(0.045)	(0.779)	(0.624)	(0.619)
$Z_{i,2001}$		$HIV2001_{c,2001}$	$HIV2001_{c,2001}$	$HIV2001_{c,2001}$
$ART_{IV,t}$		ART Cov	ART Price	ART Cost
Observations	1,064	1,064	1,064	1,064
Clusters	47	47	47	47
Kleibergen-Paap		7.44	9.60	9.45
	Ongoing Armed Conflicts (log events) - UCDP Data			
	OLS	2SLS		
	(1)	(2)	(3)	(4)
Std Growth of Population (log)	-0.206*	0.080	0.067	0.063
	(0.109)	(0.726)	(0.610)	(0.622)
$Z_{i,2001}$		$HIV2001_{c,2001}$	$HIV2001_{c,2001}$	$HIV2001_{c,2001}$
$ART_{IV,t}$		ART Cov	ART Price	ART Cost
Observations	1,064	1,064	1,064	1,064
Clusters	47	47	47	47
Kleibergen-Paap		7.44	9.60	9.45
Country f.e.	√	√	√	√
Year f.e.	√	√	√	√
Region Time Trend	√	√	√	√
HIV Trend	√	√	√	√
Primary Education	√	√	√	√
GDP p.c. (log)	√	√	√	√

Note: OLS and 2SLS estimates of β obtained from the model

$$\text{Conflict}_{c,t} = \beta \cdot \Delta\text{Population}_{c,t} + \gamma X_{c,t} + \delta_c + \zeta_t + \rho_R \cdot t + \varepsilon_{c,t}$$

The dependent variable is social conflict in country c and year t, measured as log events of conflict from the different data sets (SCAD and UCDP). The explanatory variable of interest corresponds to growth of population in country c and year t, instrumented using $Z_{c,2001} \times ART_{IV,t}$. Instrument: interaction between cross-sectional variation in the potential for ART treatment, $Z_{c,2001}$ (measured by HIV prevalence at country level, 2001), and a time-varying measure of ART expansion, $ART_{IV,t}$ (measured by global ART coverage outside Africa, or, alternatively by the global variation in the median world price of ART treatment regimens, ART Price, or by the cost of ART treatment regimens, ART Cost); the interaction term has been standardized. All empirical specifications include country fixed effects δ_c, year fixed effects ζ_t, African-subregion-specific linear time trends $\rho_R \cdot t$, and as control variables $X_{c,t}$ a linear time trend interacted with HIV prevalence in a country in 2001. All coefficients refer to standardized explanatory variables.

Table A5. Robustness: Effect of Population Growth on Conflicts controlling for Trust

	Social Violence (log events) - SCAD Data			
	OLS	2SLS		
	(1)	(2)	(3)	(4)
Std Growth of Population (log)	-0.097	-1.082**	-1.014**	-1.012**
	(0.080)	(0.527)	(0.477)	(0.477)
$Z_{i,2001}$		$HIV2001_{c,2001}$	$HIV2001_{c,2001}$	$HIV2001_{c,2001}$
$ART_{IV,t}$		ART Cov	ART Price	ART Cost
Observations	1,344	1,344	1,344	1,344
Clusters	50	50	50	50
Kleibergen-Paap		10.65	12.66	12.54
	**Ongoing Armed Conflicts (log events) - UCDP Data**			
	OLS	2SLS		
	(1)	(2)	(3)	(4)
Std Growth of Population (log)	-0.383***	-0.221	-0.314	-0.331
	(0.127)	(0.546)	(0.495)	(0.507)
$Z_{i,2001}$		$HIV2001_{c,2001}$	$HIV2001_{c,2001}$	$HIV2001_{c,2001}$
$ART_{IV,t}$		ART Cov	ART Price	ART Cost
Observations	1,344	1,344	1,344	1,344
Clusters	50	50	50	50
Kleibergen-Paap		10.65	12.66	12.54
Country f.e.	√	√	√	√
Year f.e.	√	√	√	√
Region Time Trend	√	√	√	√
HIV Trend	√	√	√	√
Trust in President	√	√	√	√
Trust in Parliament	√	√	√	√
Trust in Ruling Party	√	√	√	√

Note: OLS and 2SLS estimates of β obtained from the model

$$\text{Conflict}_{c,t} = \beta \cdot \Delta\text{Population}_{c,t} + \gamma X_{c,t} + \delta_c + \zeta_t + \rho_R \cdot t + \varepsilon_{c,t}$$

The dependent variable is social conflict in country c and year t, measured as log events of conflict from the different data sets (SCAD and UCDP). The explanatory variable of interest corresponds to growth of population in country c and year t, instrumented using $Z_{c,2001} \times ART_{IV,t}$. Instrument: interaction between cross-sectional variation in the potential for ART treatment, $Z_{c,2001}$ (measured by HIV prevalence at country level, 2001), and a time-varying measure of ART expansion, $ART_{IV,t}$ (measured by global ART coverage outside Africa, or, alternatively by the global variation in the median world price of ART treatment regimens, ART Price, or by the cost of ART treatment regimens, ART Cost); the interaction term has been standardized. All empirical specifications include country fixed effects δ_c, year fixed effects ζ_t, African-subregion-specific linear time trends $\rho_R \cdot t$, and as control variables $X_{c,t}$ a linear time trend interacted with HIV prevalence in a country in 2001. All coefficients refer to standardized explanatory variables. Controls for trust in institutions are based on survey responses from Afrobarometer (round 1 to 6) covering the period from 1999 to 2015. Observations for missing years are obtained through interpolation.

Table A6. Robustness: Effect of Population Growth on Conflicts controlling for Democracy

	Social Violence (log events) - SCAD Data			
	OLS	2SLS		
	(1)	(2)	(3)	(4)
Std Growth of Population (log)	-0.077	-1.114*	-1.029*	-1.021*
	(0.104)	(0.630)	(0.557)	(0.556)
$Z_{i,2001}$		$HIV2001_{c,2001}$	$HIV2001_{c,2001}$	$HIV2001_{c,2001}$
$ART_{IV,t}$		ART Cov	ART Price	ART Cost
Observations	1,291	1,291	1,291	1,291
Clusters	50	50	50	50
Kleibergen-Paap		8.86	10.68	10.56

	Ongoing Armed Conflicts (log events) - UCDP Data			
	OLS	2SLS		
	(1)	(2)	(3)	(4)
Std Growth of Population (log)	-0.375***	-0.166	-0.262	-0.279
	(0.118)	(0.620)	(0.532)	(0.546)
$Z_{i,2001}$		$HIV2001_{c,2001}$	$HIV2001_{c,2001}$	$HIV2001_{c,2001}$
$ART_{IV,t}$		ART Cov	ART Price	ART Cost
Observations	1,291	1,291	1,291	1,291
Clusters	50	50	50	50
Kleibergen-Paap		9.04	10.68	10.56
Country f.e.	√	√	√	√
Year f.e.	√	√	√	√
Region Time Trend	√	√	√	√
HIV Trend	√	√	√	√
Population	√	√	√	√
Polity	√	√	√	√
Polity 2	√	√	√	√
Democracy score	√	√	√	√

Note: OLS and 2SLS estimates of β obtained from the model

$$\text{Conflict}_{c,t} = \beta \cdot \Delta\text{Population}_{c,t} + \gamma X_{c,t} + \delta_c + \zeta_t + \rho_R \cdot t + \varepsilon_{c,t}$$

The dependent variable is social conflict in country c and year t, measured as log events of conflict from the different data sets (SCAD and UCDP). The explanatory variable of interest corresponds to growth of population in country c and year t, instrumented using $Z_{c,2001} \times ART_{IV,t}$. Instrument: interaction between cross-sectional variation in the potential for ART treatment, $Z_{c,2001}$ (measured by HIV prevalence at country level, 2001), and a time-varying measure of ART expansion, $ART_{IV,t}$ (measured by global ART coverage outside Africa, or, alternatively by the global variation in the median world price of ART treatment regimens, ART Price, or by the cost of ART treatment regimens, ART Cost); the interaction term has been standardized. All empirical specifications include country fixed effects δ_c, year fixed effects ζ_t, African-subregion-specific linear time trends $\rho_R \cdot t$, and as control variables $X_{c,t}$ a linear time trend interacted with HIV prevalence in a country in 2001. All coefficients refer to standardized explanatory variables. Controls for institutional quality and democracy are based on PolityIV (version 2018) covering the period from 1990 to 2018.

Table A7. Robustness: Effect of Population Growth on Conflicts controlling for Global Fund Disbursements

	Social Violence (log events) - SCAD Data			
	OLS	2SLS		
	(1)	(2)	(3)	(4)
Std Growth of Population (log)	-0.101	-0.979**	-0.885**	-0.885**
	(0.080)	(0.479)	(0.420)	(0.421)
$Z_{i,2001}$		$HIV2001_{c,2001}$	$HIV2001_{c,2001}$	$HIV2001_{c,2001}$
$ART_{IV,t}$		ART Cov	ART Price	ART Cost
Observations	1,303	1,303	1,303	1,303
Clusters	49	49	49	49
Kleibergen-Paap		12.09	14.72	14.54

	Ongoing Armed Conflicts (log events) - UCDP Data			
	OLS	2SLS		
	(1)	(2)	(3)	(4)
Std Growth of Population (log)	-0.377***	-0.166	-0.303	-0.324
	(0.128)	(0.620)	(0.442)	(0.453)
$Z_{i,2001}$		$HIV2001_{c,2001}$	$HIV2001_{c,2001}$	$HIV2001_{c,2001}$
$ART_{IV,t}$		ART Cov	ART Price	ART Cost
Observations	1,303	1,291	1,303	1,303
Clusters	49	50	49	49
Kleibergen-Paap		9.04	14.72	14.54
Country f.e.	√	√	√	√
Year f.e.	√	√	√	√
Region Time Trend	√	√	√	√
HIV Trend	√	√	√	√
Global Fund	√	√	√	√

Note: OLS and 2SLS estimates of β obtained from the model

$$\text{Conflict}_{c,t} = \beta \cdot \Delta\text{Population}_{c,t} + \gamma X_{c,t} + \delta_c + \zeta_t + \rho_R \cdot t + \varepsilon_{c,t}$$

The dependent variable is social conflict in country c and year t, measured as log events of conflict from the different data sets (SCAD and UCDP). The explanatory variable of interest corresponds to growth of population in country c and year t, instrumented using $Z_{c,2001} \times ART_{IV,t}$. Instrument: interaction between cross-sectional variation in the potential for ART treatment, $Z_{c,2001}$ (measured by HIV prevalence at country level, 2001), and a time-varying measure of ART expansion, $ART_{IV,t}$ (measured by global ART coverage outside Africa, or, alternatively by the global variation in the median world price of ART treatment regimens, ART Price, or by the cost of ART treatment regimens, ART Cost); the interaction term has been standardized. All empirical specifications include country fixed effects δ_c, year fixed effects ζ_t, African-subregion-specific linear time trends $\rho_R \cdot t$, and as control variables $X_{c,t}$ a linear time trend interacted with HIV prevalence in a country in 2001. All coefficients refer to standardized explanatory variables. Additional controls for Global Fund disbursements are included. Because the Global Fund began providing financial support in 2002, missing values prior to or after this date in our study sample are coded as 0.

References

1. Salehyan I, Hendrix CS, Hamner J, Case C, Linebarger C, Stull E, et al. Social conflict in Africa: A new database. International Interactions. 2012;38(4):503–511.

2. Sundberg R, Melander E. Introducing the UCDP Georeferenced Event Dataset. Journal of Peace Research. 2013;50(4):523–532.

3. Perriëns JH, Habiyambere V, Dongmo-Nguimfack B, Hirnschall G, et al. Prices paid for adult and paediatric antiretroviral treatment by low-and middle-income countries in 2012: high, low or just right? Antiviral Therapy. 2014;19(Suppl 3):39–47.
